# Immune Landscape in Tumor Microenvironment: Implications for Biomarker Development and Immunotherapy

**DOI:** 10.3390/ijms21155521

**Published:** 2020-08-01

**Authors:** Karim Pérez-Romero, Ramón M. Rodríguez, Amedeo Amedei, Gwendolyn Barceló-Coblijn, Daniel H. Lopez

**Affiliations:** 1 Lipids in Human Pathology, Health Research Institute of the Balearic Islands (IdISBa), Research Unit, University Hospital Son Espases, 07120 Palma, Spain; karim.perez@ssib.es (K.P.-R.); gwendolyn.barcelo@ssib.es (G.B.-C.); 2 Translational Immunology Laboratory, Health Research Institute of the Principality of Asturias, Hospital Universitario Central de Asturias, 33011 Oviedo, Spain; reprogramming@gmail.com; 3 Department of Experimental and Clinical Medicine, University of Florence, 50134 Florence, Italy; amedeo.amedei@unifi.it; 4 SOD of Interdisciplinary Internal Medicine, Azienda Ospedaliera Universitaria Careggi (AOUC), 50134 Florence, Italy

**Keywords:** tumor microenvironment, immune response, immunomodulation

## Abstract

Integration of the tumor microenvironment as a fundamental part of the tumorigenic process has undoubtedly revolutionized our understanding of cancer biology. Increasing evidence indicates that neoplastic cells establish a dependency relationship with normal resident cells in the affected tissue and, furthermore, develop the ability to recruit new accessory cells that aid tumor development. In addition to normal stromal and tumor cells, this tumor ecosystem includes an infiltrated immune component that establishes complex interactions that have a critical effect during the natural history of the tumor. The process by which immune cells modulate tumor progression is known as immunoediting, a dynamic process that creates a selective pressure that finally leads to the generation of immune-resistant cells and the inability of the immune system to eradicate the tumor. In this context, the cellular and functional characterization of the immune compartment within the tumor microenvironment will help to understand tumor progression and, ultimately, will serve to create novel prognostic tools and improve patient stratification for cancer treatment. Here we review the impact of the immune system on tumor development, focusing particularly on its clinical implications and the current technologies used to analyze immune cell diversity within the tumor.

## 1. Introduction

Our understanding of the physiological role of immunity in disease and tissue homeostasis goes hand in hand with our ability to explore the molecular complexity associated with the immune response and the intricate communication network between each of its cellular components. Recent advances in the immunology field have illuminated the way to understand diseases wherein the immune system plays a decisive role. Thus, the latest technological developments, such as mass cytometry and single-cell immunogenomics, together with system biology approaches, are enabling the analysis of the cellular composition of the immune compartment with an unprecedented level of resolution, leading to a continuous and dynamic definition of new populations of immune cells. The possibility of correlating a physio-pathological condition with a comprehensive immune signature or profile is providing new and valuable information. Hence, profiling a particular tissue thoroughly both in health and disease will help to uncover the relevant mechanisms regulating these diseases as well as identify new therapeutic targets. Importantly, the tissue-specific microenvironment state is a transversal and integrated concept that could become the starting point for personalized medicine strategies aimed at improving earlier diagnosis, prognosis, stratification, and therapy responses.

There is no doubt that the conceptualization of tumor biology as an isolated nucleus of malignant cells driven by several somatic mutations regulating cell proliferation is oversimplified and obsolete [1]. The integration of the tumor microenvironment (TME) concept, as a fundamental part of the malignization process, has revolutionized the way we study and understand cancer. The results obtained during the last decade demonstrate that neoplastic cells establish a dependency relationship with normal resident cells and, additionally, develop the ability to recruit new accessory cells that aid tumor development [2]. The resulting loss of tissue homeostasis is fundamental for disease progression, uncovering unexplored molecular targets, and providing novel opportunities for cancer treatment.

The TME is a tridimensional network formed by different subtypes of cells that actively interact with each other and with tumor cells. This complex ecosystem includes parenchymal cancer cells, tissue stromal fibroblasts/mesenchymal stromal cells, blood and lymphatic vasculature cells, and cells of immune origin [3]. In addition to the cellular component, the TME also comprises many different molecular and functional elements such as the extracellular matrix, oxygen and nutrient supply, growth factors, cytokines, and interstitial pH. In this framework, the ability of the immune system to recognize the tumor appears as an efficient antitumor strategy, since this immunosurveillance enables the immune system to seek and destroy pretumor cells, thereby preventing the development of cancer and becoming one of the most determining elements during the tumorigenic process. As described in “the three Es of cancer immunoediting” by Dunn, Old, and Schreiber [4], interaction between the immune system and tumor cells results in a dynamic adaptation of both the components and the evasive “stealth” strategy, often referred to as immunoediting, which promotes tumor evasion from immunosurveillance. During this process, tumor cells that are able to escape immune recognition, or those able to induce a tolerogenic response, are finally selected under this immunological pressure and allowed to grow and divide in an uncontrolled manner [5]. Remarkably, the concept of immunoediting was first described in the 1950s by Paul Ehrlich [6]; since then, much effort has been devoted to understanding its complex molecular and cellular networks.

Intrinsically associated with this interplay with the immune system, cancer development is accompanied by a dynamic inflammatory process [7]. Physiological inflammation triggers a series of events aimed at eliminating harmful elements and promoting tissue repair, which involves the controlled action of cells, primarily of immune origin, and soluble molecules (cytokines, chemokines, complement components, etc.). Once the causative agent is removed, a series of mechanisms involving pro-homeostatic and anti-inflammatory signals present in the tissue microenvironment resolve the inflammatory reaction. However, the persistence of the unresolved inflammatory state leads to chronic inflammation, generating an environment prone to the promotion of pretumor cells into active cancer cells. Cancer cells are able to secrete proinflammatory factors (TNFα, IFNγ, IL6, etc.) that enhance the recruitment of immune cells to the inflammation site [8], including polymorphonuclear neutrophils and eosinophils, monocytes, lymphocytes, natural killer cells (NK), and mature dendritic cells. In this inflammatory context, as well as during the early tumor stage, T lymphocytes recognize the “neoantigens” generated by the high mutational load often associated with cancer cells [9,10]. In particular, naive T cells primed in the lymph nodes migrate to the inflamed TME, wherein antigen recognition can lead to the complete removal of the tumor. Release of proinflammatory cytokines (IL2, TNFα, IFNγ, GMCSF) by Th1 lymphocytes initiate the response, triggering NK activation and neoantigen presentation by dendritic cells. Furthermore, Th (helper) 1 CD4^+^ cytokines can promote the tumoricidal role of CD8^+^ cytotoxic T lymphocytes by enhancing the production of cytolytic molecules such as perforin and granzyme in these cells, and by increasing their recruitment to the tumor site [11]. As the situation evolves, circulating monocytes infiltrate the tumor site attracted by the chemically altered environment and differentiate to tumor-associated macrophages (TAM), which also play a critical role in cancer development. These cells are highly plastic and, depending on the molecular cues present in the TME, can carry out proinflammatory or anti-inflammatory functions [12]. In a proinflammatory state, TAMs can polarize to M1 Type, and participate actively in the elimination of cancer cells. Further, TAMs can also exacerbate the acute inflammatory response, generating proinflammatory cytokines once activated by dendritic cells.

NK cells target and haunt cancer cells even in the absence of antigen specificity using a large array of stimulatory and inhibitory surface receptors. These receptors can detect aberrant traits in cancer cells, including a partial or complete loss of HLA molecules and the de novo expression of ligands that bind and activate NK cell receptors. For instance, NK stimulatory receptors like NKG2D can cause the release of perforin and granzyme as well as the induction of apoptosis in cancer cells via the release of TNFα and activation of the FAS ligand (FASL) [13]. Finally, a different subset of T cells, natural killer T cells (NKT), are involved in tumor immunosurveillance. NKT cells express an invariant T cell receptor that recognizes lipid antigens presented by a non-classical class I MHC molecule (CD1b), particularly glycerophospholipids. Within NKT cells, type I NKT cells (or invariant NKT cells) is the subtype showing antitumor activity through the secretion upon activation of a large number of cytokines, such as IL4 and IL17, TNFα and IFNγ [14].

To simplify, the lymphocyte response in the tumor is subdivided into two opposite functional poles: a Th1 response, usually associated with active tumor recognition and inflammation; and a Th2 response, characterized by an inhibition of Th1 response and mediated by the release of cytokines such as TGFβ, IL4, IL13, and IL10 by monocytes and TAM. Carcinogenesis causes macrophages to polarize to M2 type [15], while acquiring functions of tissue repair and remodeling of the extracellular matrix, and the ability to release proangiogenic (VEGF) and survival (EGF) factors. Additionally, it has been observed that the production of some cytokines, such as IL10, has an inhibitory effect on dendritic cell maturation, resulting in a lower antigenic presentation to T cells [16]. The result of this process is the generation of a TME prone to cancer development.

Hence, taking into account the complexity at the compositional and functional level of the immune system, it is clear that the mere presence of lymphoid and myeloid cell aggregates in tumors is not indicative of their participation in an antitumor effect, given that these cells may be at different activation states depending on the signals present in the TME. In this regard, it is common for tumor cells to evolve towards an immunogenicity loss and the generation of a TME that minimizes the immune system response. As the inflammation becomes chronic, processes such as matrix remodeling, angiogenesis, and lymphangiogenesis are promoted, eventually facilitating tumor growth, and invasiveness.

In summary, tumor immunity involves a highly dynamic interplay within a large variety of cell types and molecular components, which in turn is also profoundly influenced by germline and somatic genetic variations [17], microbiome (inclusive of viruses, bacteria, fungi, and parasites) [18], environmental and lifestyle (including diet, smoking habits, physical exercise) factors [19]. Although in this review we have focused on the description of the TME at the cellular and molecular level, there is no doubt that the identification of solid biomarkers to help patient stratification and to head towards a more personalized medicine, requires comprehensive and interdisciplinary approaches such as molecular pathological epidemiology (MPE) that transdisciplinary integrates molecular pathology and epidemiology [20]. While this rapidly evolving topic is beyond the scope of this review, readers are referred to a series of comprehensive reviews on this topic [20,21].

For the purpose of this article, the fundamental question is how the immune infiltrate can be studied in order to establish predictive biomarkers of tumor development, and whether this information can be used to improve clinical practice for cancer treatment. Herein we review the current knowledge on the predictive value of immune infiltration in cancer development and on the association of these cells with the response to immunotherapy.

## 2. Immunological Assessment of the TME for Cancer Prognosis

**Figure 1:**
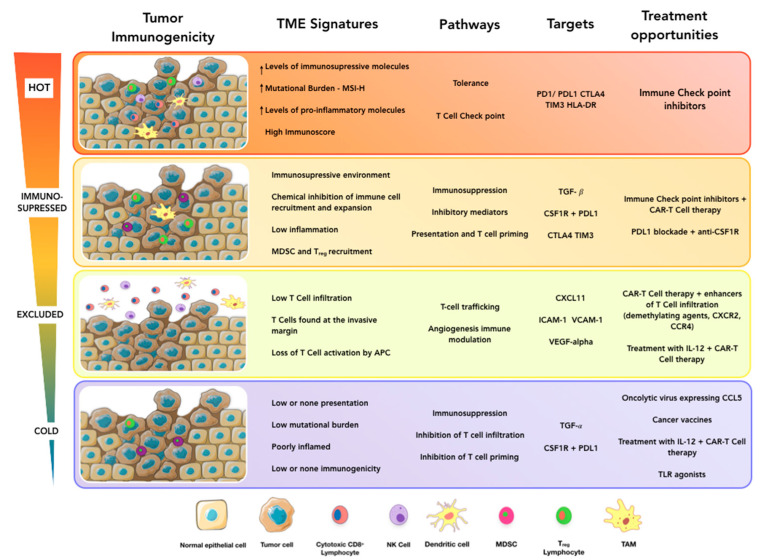
Towards a personalized cancer immunotherapy. Novel immunotherapies have demonstrated high efficacy in numerous preclinical and clinical research studies. Nonetheless, the success of these strategies is hampered by the frequent occurrence of non-responder patients. Clearly, it is essential to define the traits associated with immunotherapy-sensitive tumors in order to improve treatment success. According to lymphocyte infiltration, solid tumors are classified as hot, altered-excluded, altered-immunosuppressed, and cold. Each subtype is characterized by a particular immune signature exerting a critical impact on treatment response. Thus, the choice of immunotherapy should be guided by a previous evaluation of the immunological tumor status. In this context, hot tumors would benefit the most from immunotherapies that intensify the expansion and activity of tumor-reactive T cells, whereas cold tumors would be better treated with therapies aimed to redirect the immune response against tumoral cells. Other strategies, such as enhancing T cell trafficking or inhibiting immunosuppressive mechanisms, can help to improve immunotherapy response in some patients.

As discussed above, the immune landscape within the TME is the result of a complex interplay between many immune components, including the presence of chemoattractant factors, immunogenicity associated with the mutational burden of the tumor, alteration of antigen presentation mechanisms, or the action of immunosuppressive mechanisms (anti-inflammatory factors and immune checkpoints). Other extrinsic factors, such as vascularization and tumor location, can be critical aspects that influence the immune status of the tumor as well. In this context, it is clear that there is a causal relationship between the immune response and cancer development and, therefore, analysis of the immune contexture holds enormous clinical potential. In a seminal work, Pagès et al. showed that the immune response within colorectal cancer was associated with prolonged survival [22]. Next, Galon et al. demonstrated the prognostic value of lymphocyte infiltration in colorectal cancer (CRC) based on histopathological analysis [23]. These early initiatives centered on the immunohistochemical evaluation of CD3 and CD8 markers, and resulted in the first systematic classification of CRC based entirely on T cell infiltration [24]. Thus, according to cell density, tumors were classified as “hot” (highly infiltrated and inflamed) or “cold” (very low infiltration and not inflamed) (Figure 1) [25]. Besides, two additional altered states were defined: “excluded” (T cells found only at the invasive margin) and “immunosuppressed” (some infiltration but not inflamed). Tumors within these categories differed in their 2-year risk of relapse, which fostered confidence in the clinical value of these analyses and encouraged the development of a novel prognostic tool in cancer: the Immunoscore. This score is based on a semi-quantitative analysis of the CD8^+^/CD3^+^ ratio in both the invasive margin and the center of tumors. In colorectal cancer, it provides a better prognostic value than TNM staging [26,27,28] while its use has expanded to other cancer types, including melanoma, breast, and ovarian cancer [29]. Hence, the Immunoscore has laid the foundation stone for the systematic clinical analysis of the immune compartment present in the TME. There is no doubt that the immune contribution to cancer development is extremely complex, going far beyond what the CD8^+^/CD3^+^ ratio reflects. Current efforts should focus on the comprehensive evaluation of the immune status of the TME. In this sense, it is not surprising that, in addition to cytotoxic T cells, many other immune cell populations are also associated with overall survival among cancer patients. These cells, often organized as cellular aggregates called tertiary lymphoid structures (TLS), enable the local generation of tumor-reactive lymphocytes [30]. TLS are usually composed of a follicular zone with CD20^+^ B cells, surrounded by CD3^+^ T cell zone with lysosome-associated membrane glycoprotein^+^-(LAMP^+^-) dendritic cells. This structured organization permits a local antigen presentation of tumor antigens by dendritic cells as well as the production of effector T cells and antibody-producing plasma cells. Consequently, the presence of TLS is indicative of an active immune response against tumor cells and is, therefore, associated with a more favorable prognosis in most cancer types, including breast, lung, and ovarian cancer [31,32,33]. Surprisingly, TLS are also associated with a negative prognosis in hepatocellular cancer. This unexpected result seems to be related to the ability of cancer cells to generate immunopathological microniches within these structures [34].

In addition to TLS, the presence of some specific immune cell populations has also been connected to cancer prognosis. Macrophages are one of the most abundant and can be found dispersed within the tumor or in association with TLS. Consistent with their critical role in cancer progression, there is a clear correlation between macrophages and cancer prognosis. In general, M1 macrophage density correlates with a good prognosis, as these cells contribute to the development of a proinflammatory microenvironment, while M2 phenotypes would be associated with a poor prognosis. This correlation has been demonstrated in different cancer types such as breast, melanoma, pancreas, prostate, and lung. However, mounting evidence indicates that the specific localization of macrophages within the tumor could be a pivotal factor [35,36,37,38,39].

The prognostic potential of immune-suppressive populations in cancer, particularly the regulatory T cells (T_reg_) and myeloid-derived suppressor cells (MDSCs), has also been evaluated. Given that they may play a critical role in the modulation of the immune response (immune suppressor effect) against tumor cells, high intratumor density was predicted to be associated with a poor prognosis. In agreement with this prediction, T_reg_ infiltration is a marker of poor prognosis in lung breast cancer [40,41]; however, in colorectal and gastric cancer, it is associated with a good prognosis [42,43,44], although previously Amedei et al. have shown an enrichment of T_reg_ in tumor areas in CRC patients [45]. Moreover, conflicting results have also been reported concerning patient outcomes associated with T_reg_ infiltration, such as head and neck cancers [46,47]. This conflicting results may be related to cell plasticity [48], as several studies have shown unexpected features of this lineage depending on the microenvironment cues [49,50], expressing T cell effector functions such as the production of IL17 and IFNγ and the loss of regulatory properties [51].

Although the causes behind this disparity are not yet clear, these results suggest that the evaluation of T_regs_ independently of other immunological parameters could be insufficient to develop reliable immunological biomarkers. Meanwhile, MDSCs inhibit T cell proliferation and enhance T_reg_ recruitment and, consistently, their infiltration has also been associated with poor prognosis in several types of cancers [52].

Altogether, these results clearly indicate that different immune components can be directly related to tumor prognosis, but they also highlight the limitations of these histological strategies based on the analyses of a small series of cell surface markers. Hence, a more comprehensive approach should be taken in order to adequately assess the immune TME status and translate it into clinical practice. In recent years, the use of bulk transcriptomic data deconvolution methods, such as CIBERSORT, has enabled the infiltrated immune cell composition within cancer tissues to be explored. Thus, it is possible to obtain a more comprehensive view of the immune landscape using this procedure than histological analysis and, consequently, it could be used to define immune cell signatures with prognostic value [53]. Nonetheless, methods based on bulk analysis of tissue samples are limited by the reference profiles used to identify each cell subset. This fact is a major limitation since the transcriptional programs acquired by the immune cells are highly plastic and dependent on the cellular and molecular components within the tissue microenvironment. Fortunately, new technologies such as single-cell genomics and high dimensional flow cytometry are finally allowing the cellular complexity present within tumors to be captured. These strategies provide a simultaneous assessment of an unprecedented number of cellular parameters, which is essential to define the immune landscape. In breast carcinoma, for instance, single-cell RNA-sequencing revealed that immune cell phenotypic diversity increases within the TME compared to healthy tissue [54]. Moreover, this cellular variety does not fit well into the classical definition of discrete differentiation or polarization states. Meanwhile, T cell and macrophage populations show a variety of differentiation states, which further complicates the characterization of tumor-infiltrating immune cells. This phenotypic heterogeneity has also been observed at the protein level using mass cytometry analysis in breast cancer, demonstrating that immune cells show a wide range of expression levels of receptors associated with immune activation and exhaustion (PD1, CTLA4, HLADR, TIM3, etc.) within the TME [55]. Interestingly, some correlations between tumors and immune cell phenotypes have been detected, such as the association between the frequency of ERα^+^ tumor cells and the presence of exhaustion markers in TAM and lymphocytes that highlight the importance of a comprehensive analysis including the entire tumor ecosystem. Following a similar strategy, mass cytometry analysis of clear cell renal carcinoma showed broad expression levels of PD1, often in combination with other checkpoint proteins such as TIM3 or CTLA4 [56]. This study also highlights the limitations of the current macrophage polarization model, describing up to fifteen expression clusters in macrophages within the TME, some of which were associated with patient outcomes. In general, it is important to note that although dichotomous polarization models (Th1/Th2, M1/M2, Treg/Th17, etc.) are useful for appraising immune infiltration in cancer, high dimensional characterization of these populations by mass cytometry or single-cell RNA-seq is clearly demonstrating that immune cells are highly plastic and that they do not fit well into these models.

Overall, it is clear that due to the high level of heterogeneity and cell plasticity observed in the TME, translation of these results into clinical practice presents a massive challenge. Nonetheless, some common immune subtypes in cancer have been defined using comprehensive immunogenomic analysis [57], which includes transcriptomic, epigenomic, clinical data, and mutation analysis. Among the described phenotypes, tumors with high CD8^+^ T cell and M1 infiltration, high intratumor heterogeneity, and neoantigen burden are usually associated with an IFNγ response signature and poor prognosis. Other tumors are associated with a wound healing signature, characterized by an elevated expression of angiogenic genes, a predominance of Th2 phenotypes, and poor prognosis; or otherwise with an inflammatory signature, which is associated with elevated Th17 and Th1 infiltration but bearing a lower neoantigen burden and exhibiting better outcomes. Altogether, these analytical strategies show that systematic approaches aimed at achieving a high-resolution characterization of the immune component are undoubtedly possible, and that some common traits between tumors can be inferred and transferred to clinical practice.

Finally, it is important to note that, while the characterization of tumor infiltration is very informative, the TME is not only restricted to the tumor itself but also involves the surrounding tissues (which are dynamically affected by the pathological process) as documented in pancreatic cancer by TILS (tumor-infiltrating T cells) cloning [58] and in hepatocellular carcinoma (HCC) using single-cell RNA sequencing [59]. By analyzing the immune component in peritumoral hepatic tissue, hepatic lymph nodes, and ascites in the peritoneal cavity. These results show that immune cells in these regions exhibit distinct transcriptional states linked to tumor origin and, therefore, these populations are likely to be functionally associated with cancer development. At any rate, the clinical relevance of the information that can be extracted from the characterization of these cells is still unclear.

## 3. Evaluation of the Tumor Immune Ecosystem to Refine Current Immunotherapy

Immunotherapy is a therapeutic strategy aimed to enhance the immune response against tumor cells. There are several types of immunotherapies, some of which are well established in clinical practice, while others are currently undergoing clinical trials. Nonetheless, the response rates for immunotherapy are relatively low (10–35%) and often entail inconsistent results [60]. In this context, evaluation of the immune landscape will be critical to understand and predict the success of many of these therapies, including immune checkpoint targeting, T cell transfer therapy, and cancer vaccines.

Immune checkpoints are regulatory mechanisms that prevent exacerbated immune responses and are critically involved in self-tolerance maintenance. Hence, targeting these mechanisms can be used to enhance the expansion and activity of tumor-reactive T cells. Although many checkpoints are suitable targets for cancer immunotherapy, the PD1/PDL1 axis is the most recognized and is already in use for the treatment of both solid (melanoma, non-small cell lung cancer, renal cell carcinoma, etc.) and hematological (Hodgkin’s lymphoma) tumors. This strategy, which has proved very effective in some instances, has some important limitations because previously primed tumor-reactive T cells are required to be successful [61,62]. The fact that CD8^+^ T cell density at the invasive margin of metastasis and mutational burden in cancer cells predicts the response to PD1 blockade reinforces this observation [63,64]. Consequently, the pre-existing immunological response against the tumor seems to be the most critical element affecting the response rate to checkpoint inhibitors. This raises the question as to how the effectiveness of this strategy can be improved and whether the immunological status can be evaluated to predict response. Indeed, tumors previously classified as hot tumors, are the most likely responders to checkpoint inhibitors. In general, response to checkpoint inhibitors is better if cancer cells express high levels of immunosuppressive molecules [65,66], in the presence of high levels of inflammatory molecules within the TME [67], or when cancer cells show a high mutational burden and high-level microsatellite instability (MSI-high), caused by mismatch repair deficiency [68]. In addition, single-cell analyses have demonstrated that a specific type I interferon-driven signature in Th1 like tumor infiltrated cells responds negatively to checkpoint therapy [69], and therefore, high-resolution techniques may provide novel biomarkers to predict the response to these treatments. Taking into account that checkpoint proteins can be expressed in different levels and combinations in cancer and immune cells, a thorough analysis of the checkpoint landscape may enable the personalization of immunotherapy by targeting specific combinations of these receptors [55,56].

Conversely, cold and immunosuppressed tumors with low immune T cell infiltration and poorly inflamed would, in principle, be unsuitable candidates for immunotherapy and, especially, for immune checkpoint inhibitors, due to the very low T cell response against tumor cells. In these cases, therapies aimed at redirecting the immune response against tumor cells are likely to be more successful. Among the alternatives for these patients, chimeric antigen receptor (CAR) T cell therapy is probably the most promising. This strategy entails the genetic modification of autologous T cells with a chimeric receptor that combines an extracellular domain for recognition of a tumor antigen and the intracellular domain required for T cell activation. After infusion in the patient, CAR T cells will be able to recognize tumor cells and initiate an immune response against them. This method has been quite successful for the treatment of some hematologic malignancies, but these excellent results have not been replicated in solid tumors to date [70]. This is probably due to the immunosuppressive TME, which may prevent activation or trafficking in the tumor site of the T cell. Several combinatorial immune therapies have been proposed to overcome these difficulties, including the use of checkpoint inhibitors in combination with CAR T cells, the addition of co-stimulatory signals to intensify activation, or the coexpression of chemokine receptors to enhance tumor infiltration [71,72,73]. Another promising alternative to treat cold tumors are cancer vaccines, based on the initiation of a T cell response against tumor cells by injecting tumor-specific neoantigens, that trigger antigen-presenting cells (APC) activation and subsequent T cell priming and expansion [25]. This strategy has the potential to convert cold into inflamed tumors, although it has some limitations, such as reduced T cell repertoire in some cancer patients, low immunogenicity of the tumor-specific antigens [74] or the inherent risk of specific loss of heterozygosity affecting APC, by means of HLA haplotype loss [75].

In general, tumors that are poorly infiltrated or have an infiltration profile associated with anti-inflammatory phenotypes, such as M2 and Th2 populations, will greatly benefit from immunotherapies aimed to enhance inflammation within the TME. Thus, targeting anti- or proinflammatory pathways can be an effective approach to induce “hot” phenotypes in these tumors. For instance, targeting TGFβ, a known inducer of Th2 and M2 polarization in cancer, can increase the proinflammatory environment by enhancing NK and CD8^+^ T cell infiltration and decreasing Treg levels in preclinical models of melanoma [76,77]. Following a similar strategy, antibodies against colony-stimulating factor 1 receptor (CSF1R), the receptor of the pro M2 factor (MCSF), can directly target M2 polarization. Combined with the PDL1 blockade, this method is currently being tested to treat advanced solid malignancies [78]. The immunotherapy response can also be improved by directly stimulating proinflammatory pathways, such as those associated with Toll-like receptors (TLRs) and proinflammatory cytokines. This has been tested with a TLR signaling agonist that proved effective in cancer mouse models and is now undergoing clinical trials [79].

In addition to T cell recognition of tumor cells, T cell trafficking is another important aspect when factoring in the efficiency of immunotherapies in solid tumors. Improving T cell recruitment could enhance the efficiency of CAR T cell therapy. Furthermore, it could be critical for the treatment of tumors that are poorly infiltrated or have an “excluded” immune phenotype (i.e., T cells are in the invasive margin of the tumor but are unable to infiltrate it). Interestingly, treatment with IL12 greatly enhances NK and B cell infiltration in head and neck squamous cell carcinoma patients [80], while in mouse models, the use of the oncolytic virus expressing the chemokine CCL5 improves CAR T cell infiltration [81]. Meanwhile, CXCL9 and CXCL10 (CXCL11 in humans) are key chemokines in the recruitment of CD8^+^ T cells, which are frequently silenced in “non-inflamed” tumors by abnormal DNA hypermethylation in their genetic loci. In these tumors, the use of demethylating agents can restore chemokine production, enhancing T cell recruitment to the tumor site [82]. Finally, anti-angiogenic drugs normalize tumor vasculature and induce the upregulation of the leukocyte adhesion molecules ICAM1 and VCAM1 on tumor endothelial cells, leading to increased T cell infiltration [83].

## 4. Conclusions

Overall, it is clear that assessment of the immune status in cancer is critical to predicting the immunotherapy response and, therefore, some systematic approaches to characterize tumors at the immunological level have been proposed [84]. This is the case of the cancer immunogram, which aims to classify tumors according to immune parameters previously associated with the response to immunotherapy, including mutational load, lymphocyte infiltration, absence of checkpoints or soluble inhibitors, response to immune effectors such as IFNγ, and tumor metabolism. These and other parameters not initially included, such as macrophage and Th polarization or specific gene signatures, would provide an overview of interactions between the immune system and cancer cells that may help guide treatment choice for more personalized immunotherapy. Furthermore, novel technologies such as single-cell transcriptomics/epigenomics and high dimensional flow cytometry are rapidly expanding our ability to explore these interactions, enabling the generation of novel biomarkers associated with cancer development and therapy response. Undoubtedly, incorporation of these biomarkers in future clinical trials will provide essential information that will pave the way for more refined and personalized immunotherapy.

## Abbreviations

APC	antigen-presenting cells
CAR	chimeric antigen receptor
CCL	C-C motif chemokine ligand
CD	cluster of differentiation
CRC	colorectal cancer
CSF1R	colony-stimulating factor 1 receptor
CTLA4	cytotoxic T-lymphocyte-associated protein 4
CXCL	C-X-C motif chemokine ligand
DC	dendritic cells
DNA	deoxyribonucleic acid
EGF	epidermal growth factor
Erα	estrogen receptor alpha
FASL	FAS ligand
GMCSF	granulocyte macrophage colony-stimulating factor
HCC	hepatocellular carcinoma
HLA	human leukocyte antigen
HLADR	human leukocyte antigen DR- isotype
ICAM	intracellular cell adhesion molecule
IFNγ	interferon gamma
IL	interleukin
KLRK1	killer cell lectin-like receptor K1
LAMP	lysosome-associated membrane glycoprotein
MCSF	macrophage colony-stimulating factor
MDSC	myeloid-derived suppressor cells
MHC	major histocompatibility complex
MPE:	molecular pathological epidemiology
MSI	microsatellite instability
NK	natural killer cell
NKT	natural killer T cell
PD1	programmed cell death protein 1
PDL1	programmed cell death ligand 1
RNA	ribonucleic acid
TAM	tumor-associated macrophages
TGFβ	transforming growth factor-beta
Th	T helper cell
TIM3	T cell immunoglobulin-mucin domain 3
TLR	Toll-like receptor
TLS	tertiary lymphoid structures
TME	tumor microenvironment
TNFα	tumor necrosis factor-alpha
T_reg_	regulatory T cell
VCAM	vascular cell adhesion molecule
VEGF	vascular endothelial growth factor
